# Talking gender, ethnicity race, and change: How mothers scaffold girls' identity meaning‐making during puberty

**DOI:** 10.1111/jora.70226

**Published:** 2026-07-03

**Authors:** Rona Carter, Jessica Pitts, Joonyoung Park, Ha Bui, Julia Cross

**Affiliations:** ^1^ Department of Psychology University of Michigan Ann Arbor Michigan USA

**Keywords:** ethnic‐racial identity, gendered racial identity

## Abstract

Early adolescence is marked by intensified identity exploration, yet little research has examined how meanings of gender and ethnic‐racial identity are co‐constructed within family relationships during the pubertal transition. The present qualitative study investigated how mothers and daughters construct and negotiate meanings of gender, ethnic‐racial identity, and their intersection through dyadic conversation, and how these processes vary by daughters' pubertal status. Participants included mother–daughter dyads of Black girls (*N* = 45; *M*_*a*ge_ = 9.93, SD = .97; 69% identified as Black, 31% Black biracial) who engaged in structured identity dialogue tasks. Using reflexive thematic analysis followed by dyadic synthesis, we identified interactional processes through which identity meanings were initiated, scaffolded, negotiated, and stabilized. Across dyads, puberty functioned as a contextual intensifier, heightening emotional salience and social comparison in identity talk. In the early puberty dyads, mothers more frequently guided and defined identity meanings, whereas in the advanced puberty dyads, daughters increasingly asserted interpretive authority. Mother–daughter scaffolding operated as a relational coping process, with mothers and daughters jointly shaping how embodied change and social positioning were interpreted. Moments of misalignment and repair revealed how identity meanings were negotiated rather than transmitted. Grounded in the Phenomenological Variant of Ecological Systems Theory, findings position identity development during puberty as a relational, phenomenological process embedded within sociocultural context. By foregrounding dyadic interaction, this study extends developmental theory and highlights family dialogue as a critical microsystem linking pubertal vulnerability to identity formation.

*“Look at me, a Black girl. I'm a Black woman now but I was a Black girl. I was strong, I was independent, I was smart, I was hot‐headed and I was everything that my mom was. That's what being. For me, to be a Black girl. So, what does it mean to you?”*–Midpuberty dyad.

Puberty is a complex biopsychosocial process marked by hormonal changes that trigger the development of primary and secondary sex characteristics, such as breast development and facial hair growth (Dorn et al., 2019). Although these changes are biological, their meanings are shaped by deeply embedded sociocultural norms (Carter et al., 2024). Because gender is closely tied to assigned sex at birth, puberty often signals to adults and peers that a child is approaching adolescence and should begin to adopt behaviors aligned with socially prescribed gender roles (Carter et al., 2024; Natsuaki et al., 2015). For individuals assigned female at birth (hereafter ‘girls’), puberty marks a significant period when societal expectations regarding femininity and physical appearance become particularly prominent, many of which are rooted in historically white, Eurocentric ideals that privilege particular body types (Carter et al., 2024; Natsuaki et al., 2015). Girls whose bodies deviate from these ideals may experience heightened scrutiny, misrecognition, or marginalization, not as a matter of individual nonconformity but as a consequence of structural hierarchies governing gendered and racialized embodiment (Carter et al., 2024; Natsuaki et al., 2015). These social responses can shape girls' self‐evaluations as well as how identity meanings are negotiated in everyday interactions.

The social significance of puberty may be further intensified when girls develop earlier or later than their peers. Off‐time pubertal development has been linked to increased gender‐related pressures, peer comparison, and social evaluation (Cook et al., 2019; Helfert & Warschburger, 2013). For instance, girls who develop early may face greater self‐consciousness, feelings of alienation from their peers, or unwanted attention (Cook et al., 2019), which can complicate how they interpret bodily change and their emerging identities (Blazek & Carter, 2019; Carter et al., 2018). These tensions reflect not only biological timing but the ways girls' changing bodies are socially read and positioned within gendered and racialized hierarchies. From this perspective, puberty may function less as a discrete developmental stage and more as a contextual amplifier (Ge & Natsuaki, 2009), increasing the emotional and interpretive stakes of identity‐related experiences.

Early adolescence is therefore a critical period for identity development, not only because gender and ethnic‐racial identities are being explored, but because these explorations are embedded in relational contexts where meanings are actively constructed and negotiated in response to girls' changing bodies. Mothers, in particular, play a central role in shaping how girls interpret bodily change, social difference, and belonging through everyday conversation, guidance, and response. Although prior research has documented associations among puberty, gender identity, and ethnic‐racial identity (Umaña‐Taylor et al., 2014; Wu et al., 2023), far less is known about how identity meanings are co‐constructed in interaction, especially during the pubertal transition and among racially and ethnically diverse families.

The present study addresses this gap by examining how mothers and daughters jointly construct meanings of gender identity, ethnic‐racial identity, and their intersection through open‐ended conversation during early adolescence. Drawing on qualitative data from mother–daughter dyads of Black girls (Black and Black biracial), this study focuses on the interactional processes through which identity meanings are initiated, scaffolded, negotiated, and stabilized. Rather than treating puberty as a deterministic developmental stage, we consider pubertal status as a contextual factor that may intensify the content and dynamics of identity talk. By foregrounding dyadic interaction, this study highlights how identity development during puberty unfolds as a relational process shaped through everyday family dialogue.

## GUIDING THEORETICAL FRAMEWORK

The present study is guided by the Phenomenological Variant of Ecological Systems Theory (PVEST; Spencer, 2006; Spencer et al., 2015), a developmental framework designed to explain how individuals construct meaning and identity within socioculturally stratified contexts. PVEST emphasizes that development unfolds through individuals' subjective interpretations of lived experience, particularly in contexts marked by vulnerability, stress, and unequal access to social power. Rather than conceptualizing identity as a static trait or purely cognitive construct, PVEST views identity as an emergent process, shaped over time through repeated meaning‐making and coping with responses to contextual demands.

PVEST is particularly well suited for understanding identity development during puberty, a period that introduces new biological, social, and emotional challenges while simultaneously increasing youths' visibility within gendered and racialized social systems. From a PVEST perspective, puberty constitutes a contextual vulnerability rather than a deterministic developmental stage. Bodily changes do not inherently produce identity outcomes; instead, their developmental significance depends on how youth interpret these changes and how others respond to them within family, peer, and cultural contexts. These interpretations are shaped through ongoing interaction and can become stabilized into identity meanings over time.

Within this framework, maternal socialization operates as a key ecological and relational resource, shaping how girls interpret pubertal change, social difference, and belonging. PVEST highlights coping as a central developmental mechanism, encompassing both protective and reactive responses to stress. In the present study, mothers' conversational scaffolding through prompting, expansion, refinement, and repair functions as a form of relational coping support, providing interpretive tools that girls may draw upon as they navigate emerging identity‐related challenges. At the same time, daughters are active participants in these exchanges, shaping meaning through engagement, resistance, or reinterpretation. This relational emphasis aligns with PVEST's focus on development as transactional and phenomenological, rather than unidirectional.

To attend to how systems of power shape vulnerability and meaning‐making, the present study is informed by intersectionality as a critical analytic lens (Collins & Bilge, 2020; Crenshaw, 1991, 2017). Intersectionality emphasizes that social categories such as race and gender do not operate independently, but intersect within systems of inequality that structure lived experience. Importantly, intersectionality is not treated here as a theory of individual identity development, but as a framework for understanding how structural conditions shape the contexts in which identity meanings are constructed.

This lens is essential for interpreting how pubertal development is experienced differently across girls from diverse ethnic‐racial backgrounds. For example, Black girls are disproportionately subjected to adultification bias, whereby they are perceived as older, less innocent, and more sexually mature than their peers (Epstein et al., 2017). Such perceptions can intensify scrutiny of Black girls' bodies during puberty and shape the meanings that emerge in identity‐related conversations. Similarly, research suggests that girls with bicultural or multiracial backgrounds may engage in more complex identity negotiations, particularly when navigating contexts that differentially value aspects of their racialized and gendered selves (Sparks et al., 2023).

By integrating PVEST with an intersectional analytic lens, the present study conceptualizes identity development during puberty as a relational, contextually embedded process, shaped through dyadic interaction and situated within systems of gendered and racialized power. This approach allows us to examine not only what identity meanings girls articulate, but how those meanings are co‐constructed, negotiated, and stabilized through everyday conversations with mothers during a period of embodied change. This framing also shifts attention away from maternal socialization as a one‐directional transmission of values and toward identity meaning‐making as a transactional process. Mothers may initiate, clarify, and scaffold identity meanings, but daughters also shape the interaction through hesitation, elaboration, resistance, humor, and refusal. Thus, the dyad becomes the developmental site through which identity meanings are not simply delivered but negotiated.

### Identity meaning‐making during early adolescence: Gender, ethnicity, and race

Early adolescence is a developmental period marked by heightened attention to the self and increased sensitivity to social evaluation. During this time, youth begin to actively interpret what their social identities mean for who they are, how they are perceived, and how they belong within their social worlds. For Black girls in particular, identity meaning‐making often unfolds across multiple, intersecting dimensions, including gender, ethnicity, and race. These dimensions do not develop independently; rather, their meanings are constructed through lived experience, social interaction, and interpretation of culturally embedded expectations.

Research on ethnic‐racial identity (ERI) development has consistently demonstrated that adolescents' engagement with questions of race and ethnicity is associated with important developmental outcomes, including self‐esteem, academic motivation, and resistance to risky behaviors (Rivas‐Drake et al., 2014; Sladek et al., 2020; Wantchekon & Umaña‐Taylor, 2021; Zapolski et al., 2019). ERI exploration, in particular, is salient during early adolescence, as youth begin to seek information about their racial or ethnic background, engage with cultural traditions, and interpret experiences of inclusion or exclusion (Huguley et al., 2019; Umaña‐Taylor et al., 2014). Importantly, this process is not uniformly protective. For some youth, greater exploration is associated with increased exposure to discrimination and heightened emotional distress, illustrating that identity work can simultaneously serve as a resource and a source of vulnerability (Umaña‐Taylor et al., 2015).

From a PVEST perspective, these patterns underscore that identity development reflects phenomenological meaning‐making in response to contextual demands rather than linear progression toward positive outcomes. How youth interpret and integrate experiences related to race and ethnicity depends on the social meanings attached to those experiences and the relational supports available to them. Mothers are among the most influential of these supports, shaping how youth understand, evaluate, and respond to identity‐relevant experiences (Gonzales‐Backen et al., 2023).

Compared to ERI, gender identity development has received less empirical attention during early adolescence, particularly among girls of color. Gender identity encompasses beliefs about belonging, contentedness, and perceived pressure to conform to gender norms (Egan & Perry, 2001), all of which are socially mediated. Existing research suggests that girls begin to grapple with gendered expectations in childhood, well before adolescence, as they encounter cultural messages about appearance, behavior, femininity, and social roles (Brown et al., 2011; Skinner et al., 2018; Thomas & King, 2007). Media portrayals, peer interactions, and adult responses contribute to how girls come to understand what it means to be a girl and whose bodies are valued or scrutinized (Gordon, 2008).

For Black girls, gender identity meaning‐making is frequently shaped by racialized and gendered social experiences. Qualitative research highlights how Black girls, in particular, experience hypervisibility and surveillance of their bodies, hair, and appearance, often through microaggressions from peers and educators (Gadson & Lewis, 2022). Such experiences have been linked to increased emotional distress and hostility, suggesting that gendered embodiment is interpreted within racialized systems of meaning (Grower et al., 2021). These findings point to the importance of examining gender identity not as an individual attribute, but as a socially interpreted and relational process.

### Gendered ethnic‐racial identity and intersectional meaning‐making

An additional layer of complexity emerges when gender and ethnic‐racial identities intersect. Gendered ethnic‐racial identity refers to how meanings of gender are shaped by racialized and cultural contexts, and vice versa (Brown et al., 2017). Rather than representing separate domains, these identities are often experienced as intertwined, particularly during early adolescence when social expectations intensify and bodily changes become more visible. Research illustrates that mothers often communicate gendered ethnic‐racial meanings in ways that reflect structural realities and collective histories. For example, Black mothers have been found to encourage their daughters to cultivate pride, academic ambition, and caution in romantic relationships—messages shaped by gendered racism and concerns for safety (Thomas & King, 2007). Intersectionality provides a critical lens for understanding these processes by emphasizing how systems of power shape lived experience. Importantly, in the present study, intersectionality is not treated as a theory of individual development, but as a framework for understanding the structural conditions within which identity meanings are constructed. This lens clarifies why identity work may be experienced differently across girls from diverse ethnic‐racial backgrounds, even when navigating similar developmental transitions.

### Puberty as a contextual intensifier of identity meaning

Puberty represents a pivotal moment in early adolescence, not only because of biological change, but because of its social and symbolic significance (Carter et al., 2024). As girls' bodies change, they become increasingly visible within gendered and racialized systems of evaluation, altering how they are perceived and how they interpret themselves (Natsuaki et al., 2015). These changes unfold within sociocultural contexts that assign meaning to bodily development, timing, and appearance (Natsuaki et al., 2015). Despite the centrality of puberty to adolescent development, relatively little research has examined how pubertal timing intersects with identity meaning‐making processes, particularly at the intersection of gender and ethnicity race (Cavanagh, 2004; Hamlat et al., 2014; Seaton & Carter, 2020). Existing evidence suggests that early or off‐time pubertal development can amplify vulnerability, especially for Black girls whose bodies are already subject to racialized scrutiny (Carter et al., 2017; Seaton & Carter, 2019; Townsend, 2002). For example, Townsend (2002) found that Black girls with less developed ethnic‐racial identities reported greater sexual risk‐taking following menarche, while Carter et al. (2017) demonstrated that pubertal timing moderated associations between public regard and internalizing symptoms among Black adolescents. These findings suggest that puberty may function as a contextual amplifier, intensifying the emotional and interpretive stakes of identity‐related experiences rather than directly producing identity outcomes. From a PVEST perspective, puberty introduces new vulnerabilities that require interpretive and coping responses, the meanings of which are shaped through social interaction.

### Maternal socialization as a relational context for identity meaning‐making

Mothers play a critical role in shaping how girls interpret identity‐relevant experiences, particularly during early adolescence when questions of belonging and self‐understanding are heightened. Prior research on maternal ethnic‐racial socialization has identified key message domains, including cultural socialization, preparation for bias, and affirmation (Hughes et al., 2006). These messages are associated with adolescents' ERI development and well‐being, though their effects vary depending on developmental timing and context (Gonzales‐Backen et al., 2023). However, much of this literature has focused on the content of socialization messages rather than the interactional processes through which meanings are constructed. From a PVEST‐informed perspective, maternal socialization is best understood as a relational process in which mothers and daughters jointly interpret experiences, negotiate meanings, and respond to contextual demands. Gender socialization operates similarly, with mothers shaping daughters' understandings of femininity through modeling, instruction, and conversational engagement (Hilliard & Liben, 2014; Rittenour et al., 2014).

Importantly, maternal gender socialization is embedded within racialized and cultural contexts. Black mothers, for example, often emphasize strength, pride, and resilience in response to gendered racism (Leath et al., 2023). These practices reflect gendered ethnic‐racial socialization, through which girls learn how race, gender, and power intersect in their lives (McHale et al., 2006; Williams & Lewis, 2021). Understanding how these meanings are co‐constructed in conversation is essential for capturing the relational and embodied nature of identity development during puberty. By focusing on dyadic interaction rather than isolated messages or outcomes, the present study seeks to illuminate how identity meanings are initiated, scaffolded, negotiated, and stabilized within mother–daughter relationships during a period of heightened developmental vulnerability.

### The Current Study

Although research on puberty, gender, and ethnic‐racial identity has expanded in recent years, relatively little empirical work has examined how these domains are jointly interpreted and negotiated in everyday interaction. Much of the existing literature focuses on identity outcomes or message content, leaving less attention to the processes through which identity meanings are constructed during the pubertal transition. The present study addresses this gap by examining how mothers and daughters co‐construct meanings of gender, ethnic‐racial identity, and their intersection through open‐ended conversation during early adolescence.

More importantly, this study conceptualizes puberty not as a deterministic developmental stage, but as a contextual vulnerability that may intensify the emotional and interpretive stakes of identity talk. Drawing on qualitative data from mother–daughter dyads of Black and Black biracial girls, we focus on the interactional processes through which identity meanings are initiated, scaffolded, negotiated, and stabilized. Rather than comparing identity content across groups, the analytic emphasis is on how meaning unfolds within dyadic interaction and how pubertal status shapes conversational dynamics. By centering mother–daughter dialogue, this narrative‐based, dyadic approach captures identity development as a relational and phenomenological process, shaped through interaction within broader sociocultural contexts. This approach enables an examination of not only *what* identity meanings Black girls articulate, but *how* those meanings are co‐constructed, contested, and resolved in conversation during a period of embodied change.

Guided by the PVEST, the present study addresses the following research questions: RQ1. How do mothers and daughters jointly construct meanings of gender, ethnic‐racial identity, and gendered ethnic‐racial identity through dyadic conversation during early adolescence? RQ2. What interactional processes (e.g., initiation, scaffolding, negotiation, and repair) characterize mother–daughter identity talk across these identity domains? RQ3. How does pubertal context shape the emotional salience, content, and interactional dynamics of identity meaning‐making within mother–daughter conversations? RQ4. Whose interpretations tend to hold within dyadic identity conversations, and how do patterns of conversational authority and voice vary across pubertal contexts and identity domains?

## METHOD

### Participants

Forty‐five mother‐daughter dyads of Black girls from an ongoing study on identity exploration during the pubertal transition and the dynamics of mother‐daughter relationships at puberty were included; 67% of the mothers identified as Black, 27% White, 4% Bi/Multi‐racial, and 2% either Latine, American Indian/Alaska Native, or Asian. The sample consisted of daughters aged 9–12 years (*M*_*a*ge_ = 9.93, SD = .97); 69% identified as Black and 31% Black biracial. Daughters who identified as biracial all identified Black as one of their racial identities; additional racial identities included 43% White, 36% Native American, 14% Latine, and 7% Asian. 78% had an older sibling. 58% of the mothers were single/never married, 44% were employed full‐time (*M =* family income of $40,000), and 49% had attained some college education.

### Procedure

Mothers of daughters aged 9 to 12 were recruited from local schools and through Craigslist and popular family gathering places, such as doctors' offices, community centers, and churches, in Midwestern cities in the United States. The mothers and youth participants completed a series of questionnaires, including the Pubertal Development Scale (Petersen et al., 1988). These questionnaires were administered on computers using Qualtrics (2025).

Instructions for each questionnaire were read aloud, and examples were provided to the youth. If necessary, one of the study's research assistants would read the questionnaires aloud to the youth participants; during these instances, the youth followed along and completed the forms independently, ensuring that the assistants did not view the participants' responses to minimize any potential demand effects.

Before completing the study questionnaires, both the youth and their mothers participated in a reading task. In this task, mothers were instructed to read five sections (i.e., skin changes, breast development, menstruation, height changes, and hair growth) from the puberty book, *The Care and Keeping of You 2: The Body Book for Older Girls* by Cara Natterson (2012), to their daughters. After the reading, mothers posed three open‐ended questions to their daughters: “What does it mean to you to be a girl?” (gender identity), “What does it mean to you to be [insert ethnicity/race]?” (ethnic‐racial identity) and “What does it mean to you to be a [insert ethnicity/race] girl?” (gendered ethnic‐racial identity). Mother‐daughter interactions during this task were videotaped and transcribed for analysis. To better understand the influences that shape our research approach and findings, we invite you to review our positionality statement; see Table 1.

**TABLE 1:** Positionality Statement of Authors.

Team member	Salient identities	Key assumptions, biases, or blind spots	Methods used to account for key assumptions, biases, and blind spots
1st author	Black, dark‐skinned, cisgender woman, social class, and education‐related privileges related to being an academician.	A critical stance toward systems, ideologies, and practices that reproduce whiteness, white supremacy, and other forms of dominance. Similarly, a bias against expressions of traditional or hegemonic masculinity that perpetuate gendered oppression or silence vulnerability.	Recruited a racially diverse research team with varied approaches and commitments. Welcomed feedback about blind spots and biases. Remained attentive to how bias may constrain my openness to alternative viewpoints or experiences that do not align with liberation‐centered paradigms
2nd author	Black cisgender woman, education‐related privileges as a doctoral student	Working under expectations that individuals from opportunity‐deserving communities create their own ways of knowing.	Welcomed feedback about blind spots and biases, and updated the analysis accordingly
3rd author	East Asian cisgender woman with privileges as an international researcher in the United States.	Expecting diverse patterns of findings across ethnic‐racial groups, yet still holding the bias that families from diverse backgrounds may show similar results, given the universality of pubertal transition.	Welcomed feedback about blind spots and biases, and updated the manuscript accordingly
4th author	Southeast Asian woman, with education‐related privileges as an international doctoral student in the United States.	Bias against individuals with class, race, and citizenship status‐related privileges.	Welcomed feedback about blind spots and biases and updated the analysis accordingly.
5th author	Latina cisgender woman with privileges to be an undergraduate student at a university.	Working under the premise that members of marginalized communities are the real experts of their lived experiences.	Welcomed feedback about blind spots and biases and proceeded accordingly.

### Measures

#### Pubertal status

The Pubertal Development Scale (PDS; Petersen et al., 1988) was used to assess pubertal status. It is a 5‐item self‐rating scale designed to assess the extent to which children experience pubertal growth in several domains during the past 12 months. Youth respond 1 (have not begun) to 4 (development completed) to all items. Girls rated their body hair development, growth spurt, skin changes, development of breasts, and the occurrence of menarche. Following established scoring procedures, responses were combined to create a categorical pubertal status variable reflecting stage of development. The original scoring scheme produced five categories: 1 = prepubertal, 2 = early pubertal, 3 = midpubertal, 4 = late pubertal, and 5 = postpubertal.

Puberty category scores were computed using reported levels of body hair growth and breast development in combination with menarcheal status. Classification criteria were as follows: prepubertal (composite score = 2 and no menarche), early pubertal (score = 3 and no menarche), midpubertal (score > 3 and no menarche), late pubertal (score ≤ 7 with menarche), and postpubertal (score = 8 with menarche). For analytic purposes in the present study, categories were collapsed to create three broader developmental stages. The prepubertal and early pubertal groups were combined and labeled beginning puberty, the midpubertal category was retained as midpuberty, and the late and postpubertal categories were combined and labeled advanced puberty. Past research has established satisfactory predictive validity for the PDS using a physical exam (significant correlations ranging from 0.61 to 0.67; Brooks‐Gunn et al., 1987; Robertson et al., 1992). Bond et al. (2006) reported moderate agreement between the PDS and self‐rated Tanner Stage (*κ* = 0.50). Petersen et al. (1988) reported internal consistency estimates (coefficient alphas) for girls ranging from 0.76 to 0.83. In this sample, the coefficient alpha was .65.

### Analysis plan

We employed a data‐driven reflexive thematic analysis approach (Braun & Clarke, 2013) to explore how Black girls construct meaning around their gender, ethnic‐racial, and gendered ethnic‐racial identities during the pubertal transition. This analytic approach was well‐suited to our goal of identifying nuanced, inductively generated themes across identity domains and understanding how these meanings were shaped through mother‐daughter dialogue and the pubertal transition. The analysis proceeded in two complementary phases. In the first phase, we conducted thematic coding to identify recurring identity‐relevant meanings within maternal and daughter talk across three identity domains (gender, ethnic‐racial, and gendered ethnic‐racial identity). The unit of analysis in this phase was the individual response within the mother–daughter dyad.

In the second phase, these validated themes were reorganized dyadically to examine how identity meanings were initiated, expanded, negotiated, and stabilized through interaction. This phase involved an interpretive synthesis of previously coded material rather than new coding, with the goal of foregrounding interactional processes and relational sequencing. Thus, the dyad functioned as the analytic lens for presentation and interpretation, while thematic coding remained the empirical foundation of the analysis.

To examine developmental variation, dyads were grouped by daughters' pubertal status using scores from the Pubertal Development Scale (PDS; Petersen et al., 1988): Beginning puberty (*n* = 8; *M*age = 9.19, SD = .40), Mid‐puberty (*n* = 29; *M*age = 9.98, SD = .87), and Advanced puberty (*n* = 8; *M*age = 10.75, SD = 1.17). Pubertal status was treated as a contextual moderator of identity meaning‐making, rather than a deterministic developmental stage.

#### Coding process

The analytic process followed Braun and Clarke's (2013) six phases of thematic analysis: (1) Familiarization: The primary coder reviewed the full transcripts of mother‐daughter conversations, conducted following a structured identity dialogue task embedded in a puberty‐focused reading activity, (2) Initial coding: Using NVivo 12 (Lumivero, 2018), the primary coder developed preliminary codes by closely analyzing responses from 15 dyads. These codes were informed by participants' language, context, and patterns across conversations, (3) Memoing and reflexive journaling were used throughout the process to capture analytic insights, identity‐relevant concepts, and theoretical tensions, particularly in relation to embodiment, stereotype awareness, and intersectionality, (4) Theme development: Codes were iteratively grouped into broader conceptual themes through analytic discussion between the primary coder and the first author. Themes were defined not only by frequency but also by conceptual salience and diversity of expression across groups, (5) Review and refinement: Themes were reviewed and refined to ensure they captured meaningful patterns across racial, ethnic, and identity domains, and (6) Validation and reliability check: To assess thematic reliability and ensure coding consistency, a second, independent coder applied the final codebook to 15 additional mother‐daughter dyads. Interrater agreement was calculated for each identity domain using Cohen's Kappa: Gender identity: *κ* = .76 to .90, Ethnic‐racial identity: *κ* = .82 to .96, and Gendered ethnic‐racial identity: *κ* = .74 to .88. These reliability estimates apply to the foundational thematic coding that undergirds all subsequent analyses.

Following thematic validation, results were reorganized to highlight interactional processes within mother–daughter dyads. Rather than treating maternal and daughter themes as separate analytic products, this synthesis examined how their contributions functioned together across phases of interaction (e.g., initiation, scaffolding, negotiation, and repair). This reorganization represents a second‐order analytic interpretation of the validated themes rather than a new coding procedure. Selected conversational excerpts are presented as analytic vignettes to illustrate interactional patterns identified across the dataset. These vignettes are not treated as independent cases but as representative examples grounded in the broader thematic structure. To reflect the study's developmental focus, quoted excerpts are identified by daughters' pubertal status rather than chronological age.

## RESULTS

Results are organized around five dyadic processes: (1) maternal entry points and invitations that initiate identity talk, (2) scaffolding processes that expand and refine meanings, (3) negotiation and tension when meanings misalign, and (4) patterns of authority and voice that determine whose meanings ultimately stabilize within conversation. We conclude with results that highlight how puberty serves as a contextual intensifier. Illustrative excerpts are drawn from the three identity prompts (gender, ethnic‐racial, gendered ethnic‐racial) and are noted by pubertal status group (beginning, mid, and advanced).

### Initiating identity talk: Maternal entry points and invitations

In discussions about identity, mothers often assumed the lead by introducing structured prompts that encouraged daughters to reflect while also maintaining direction over the conversation. These prompts typically centered on recognizable and concrete aspects of identity, offering daughters accessible entry points for engagement. In this way, early identity talk often began through guided prompts that drew on cultural familiarity and maternal scaffolding. At this stage, identity meanings were not yet negotiated or contested; rather, they were elicited, stabilized, and affirmed within the interaction. Through this process, mothers appeared to create an interpretive starting point for daughters, laying the groundwork for more complex meaning‐making as development unfolds.

For example, in one beginning‐puberty dyad, the mother initiated the task by asking, “*What does it mean to you to be a girl*?” The daughter responded with uncertainty, “*I don't know*,” accompanied by tentative laughter. Rather than leaving the ambiguity unresolved, the mother quickly reframed the question by narrowing its focus: “*What's special about being a girl versus being a boy*?” This shift signaled that an answer was expected and that the task could be approached through recognizable contrasts.

As the exchange continued, the mother guided the discussion toward concrete and socially familiar dimensions of gender, including appearance (“*having long hair*”), clothing (“*wearing dresses*,” “*jewelry*”), and biological difference (“*having a baby*,” “*using the breast to feed the baby*”). Each prompt narrowed the interpretive space, transforming an open‐ended identity question into a series of bounded options. The daughter's responses mirrored this structure, remaining brief and confirmatory (“*Yeah*,” “*That's it*”), suggesting participation without elaboration.

A similar pattern emerged when the conversation turned to ethnic‐racial identity. The mother listed ancestral categories and asked what it meant to be African American and Greek. The daughter oriented to a familiar entry point, describing Greek identity through food (“*we have yummy food*,” “…*gyros*”). The mother affirmed this response before extending the discussion to African American history and culture, again emphasizing recognition rather than interpretation.

When asked about gendered ethnic‐racial identity, the mother asked, “*What does it mean to be an African American girl and a Greek girl*?” The daughter returned to previously established anchors, referencing Greek goddesses and food, and offering a brief affective evaluation (“*it feels good*”). The mother affirmed the response (“*There is no wrong answer*”), bringing the exchange to a close without further prompting for integration.

### Scaffolding meaning through interaction: Expansion, refinement, and alignment

Once identity talk was initiated, mothers shaped daughters' interpretations through three recurring scaffolding processes: expansion, refinement, and alignment. Expansion involved mothers adding emotional, developmental, or cultural context that broadened what identity could mean. For example, one mother in the advanced puberty dyad extended the meaning of gender beyond a simple label by referencing shared resources: “*What did we learn about in the book?*” Then, she normalized developmental variability, explaining, “*We all grow differently. No girl grows the same. We all have our own way of growing*.” Through these moves, mothers framed pubertal change as a shared developmental process rather than a private bodily disruption.

Refinement occurred when mothers encouraged daughters to move beyond naming identity categories toward articulating evaluation, pride, or personal meaning. For example, during ethnic‐racial identity talk, one mother (mid‐puberty dyad) asked, “*How do you feel as a person? To have, to be blessed with all of those different ethnicities?*” This prompt shifted the task from identification to interpretation, positioning multiracial identity as a source of value and reflection. Alignment appeared when mothers linked daughters' identities to broader cultural narratives and collective belonging. For instance, one mother (mid‐puberty dyad) reminded her daughter, “*There's a whole month…dedicated to us*”, situating identity within shared historical recognition. In discussions of gendered ethnic‐racial identity, alignment also included invitations for integrative reflection: “*Now what does it mean to be a mixed girl? It's different than being just an African American girl or just a German French girl*.” (mid‐puberty dyad). Other prompts connected identity to contemporary social location: “*You a Black girl living in America right now. What does that mean to you? How does that make you feel?*” (mid‐puberty dyad).

Across dyads, daughters varied in how they took up these scaffolds. Some responses remained brief and label‐based (e.g., “*I don't know*”), whereas others reflected increasingly elaborated interpretations as scaffolding intensified. For example, one daughter expressed pride in racial identity, “*Black people are perfect*,” (advanced‐puberty dyad), while another articulated more complex gendered meaning, “*It means I'm fabulous, but it's also hard because I think guys are much luckier”* (beginning puberty dyad). Across prompts, gendered ethnic‐racial identity talk often required dyads to integrate meanings that had been discussed separately in relation to gender and ethnicity race. These exchanges were more likely to involve comparison, appearance, social treatment, and belonging, suggesting that gendered ethnic‐racial identity carried a more integrative and socially evaluative demand than either identity domain alone.

### Moments of misalignment, resistance, and repair

Not all identity conversations unfolded smoothly. Across dyads, identity talk sometimes produced moments of misalignment between mothers' framing and daughters' readiness, particularly when topics carried greater emotional weight (e.g., discrimination or body evaluation). Rather than escalating into overt conflict, these moments typically took the form of subtle interactional patterns: mothers pressed for elaboration, daughters constrained the conversational scope, and the dyad resolved the mismatch through repair and closure.

One beginning‐puberty dyad illustrates this pattern across all three prompts. When asked what it means to be a girl, the daughter initially framed gender in terms of a broad difference claim, “*I'm different from everybody else*”, which the mother treated as incomplete and prompted for elaboration, “*And what else?*” The daughter reiterated difference as a generalized life experience, “*different things in life happen to me but not to other people*,” and the mother attempted to refine the meaning toward embodiment, “*To you or to your body*?” The daughter narrowed the claim to the body, “*To my body*”, but signaled conversational closure through brief responses and nonverbal confirmation as the mother continued to probe, “*Mhm. That's it?”; “You sure?*” The exchange concluded when the mother accepted the limit and formally ended the discussion, “*Okay, we're done with that*.”

A similar pattern emerged in the ethnic‐racial identity prompt. The daughter initially framed African American identity through categorical difference, *“some people are…not African American,”* and then geographic distinction, “*I come from a different state*.” The mother gently redirected the frame toward heritage, “*You come from Michigan, a different heritage you mean?*” After the daughter's minimal affirmation, “*Mhm*”, the mother again pursued affective meaning, “*What does it mean…to be Black? How do you feel?*” The daughter responded with low‐affect closure, “*Fine*”, prompting further probes by the mother, “*What do you mean by fine?*” and a higher‐stakes question about discrimination, “*Do you feel like people treat you differently?*” Rather than entering that frame, the daughter redirected the discussion to a normative principle, “*People should be treated the same*” When the mother attempted to anchor the question in personal experience, “*So you feel like people treat you the same?*” the daughter offered partial qualification, “*Sometimes*” before affirming positive identification when asked if she liked being African American, “*Mhm!*”.

Tension became most visible in the gendered ethnic‐racial prompt, where the mother attempted to move from identity labeling to relational comparison. The daughter began with a firm assertion of stability, stating that being an African American girl meant “*I don't wanna be changed*.” When the mother introduced a comparison to a White peer, the daughter identified the friend but rejected the premise of difference, “*No” “Nuh uh*”. The mother continued to seek clarification: “So you both get treated the same way?” “So it's no difference?” receiving brief affirmations and headshakes that limited elaboration. The exchange concluded when the mother accepted the daughter's framing and ended the conversation with “*Alright*.”

Across these segments, the daughter's responses did not simply reflect avoidance; rather, they suggested an effort to contain meaning at a manageable level (difference as embodied change, identity as heritage, fairness as principle, sameness in peer relationships). In contrast, the mother consistently attempted to deepen interpretation through refinement and experiential probes. Repair occurred when mothers shifted from continued pursuit to acceptance and closure, preserving relational safety and allowing the interaction to move forward without rupture. Together, these moments illustrate how dyadic identity conversations involve negotiated boundaries around vulnerability, particularly when discussions touch on treatment, comparison, or discrimination.

### Whose meaning holds? Patterns of authority and voice in dyadic identity talk

Across dyads, identity meanings were not always jointly negotiated. In some interactions, conversational authority became contested, revealing whose interpretations ultimately structured the exchange. One mid‐puberty dyad illustrates how authority could be exercised through persistence and elaboration, even when daughters signaled disengagement. When asked what it means to be a girl, the daughter initially responded with uncertainty, “*I don't know*”, followed by a concise biological definition, “*To have kids*”. Rather than accepting this response, the mother expanded and reframed the meaning, layering biological, social, and ideological claims, “*To have kids and to carry a kid…men are told that they can do math and science…and girls are told that they can't. But to me, to be a girl means you can do whatever you want*.” The daughter attempted to close the exchange, “*Okay, next question*”, indicating that her contribution was complete.

Despite this cue, the mother persisted, explicitly asserting interactional authority, “*Well you have to answer something*” and continuing to elaborate meanings related to femininity, appearance, and choice, “*you can wear dresses and be pretty…if you want to*” The daughter's replies, “*Sure, Okay next question*” functioned as minimal compliance, acknowledging the mother's speech without extending the interpretation. When the mother added an appearance‐based claim, “*you can have curly hair*”, the daughter briefly contested it, “*Boys can have curly hair*”, reclaiming interpretive authority before again attempting to move the task forward.

A similar pattern emerged during the gendered ethnic‐racial identity prompt. After the daughter defined being African American as “*mixed*” and described identity through cultural exposure, “*learn about different things…eat different foods*”, the mother reposed the question to emphasize gendered difference, “*Now what does it mean to be a mixed girl?*” The daughter resisted the reframing, “*Didn't I just answer this question? I don't know*”, while the mother anchored identity meaning in racialized appearance, “*you look like a white girl but you're mixed with African American*”. The daughter responded with a detailed numerical description, “*75% white and 25% blac*k”, reclaiming authority through factual clarification rather than affective interpretation. The exchange ended not through shared agreement but through termination, after the daughter expressed boredom, “*Can we be done? I'm bored*”. The mother's final response, “*Alright,”* marked a relinquishing of conversational authority without integrating the daughter's interpretation. In this interaction, meaning is stabilized through closure rather than consensus, illustrating how authority in dyadic identity talk can persist, fracture, or dissolve depending on relational endurance.

In contrast, some dyads reflected smooth alignment, where maternal guidance was accepted and meanings stabilized without contestation. In one such interaction (beginning puberty dyad), the daughter defined being a girl through embodied developmental change, “*I'll have a period and puberty*.” The mother refined these meanings through confirmatory prompts, “*What about your body?”; “…you know your body will change before boys do?*”, which the daughter consistently affirmed with “*Yeah*.” When asked whether she liked being a girl, the daughter responded positively, allowing the exchange to stabilize without tension.

A similar pattern appeared in discussions of ethnic‐racial and gendered ethnic‐racial identity. The daughter initially framed African American identity in terms of visible difference, “*I'll look different*”, which the mother refined by naming specific features (skin color, hair texture, and eye color). The daughter's responses remained concrete but consistently affirmed the mother's framing. When the daughter signaled completion (“*No*”), the mother accepted the boundary (“*Okay*”). The final prompt about being an African American girl followed the same trajectory; the daughter described differences in appearance and experience, the mother summarized, and the interaction closed with mutual acceptance (“*Yeah? Okay*.”). In this dyad, identity meanings are stabilized through alignment rather than contestation.

Together, these exchanges illustrate that authority in dyadic identity talk is dynamic rather than fixed. Mothers often guided interpretation through expansion and persistence, yet daughters could assert control through resistance, procedural redirection, or withdrawal. Whose meaning ultimately “held” depended not only on developmental readiness but also on interactional stamina, emotional investment, and the ability to sustain engagement. Whereas some dyads resolved authority through persistence or disengagement, others reached stabilization through alignment, with maternal guidance accepted and meanings contained through shared agreement.

### Puberty as a conversational intensifier: Contextual, not deterministic

Across dyads, puberty did not simply introduce new identity content; it altered the stakes of identity conversation itself. As girls' bodies became more visibly marked by pubertal change, questions about gender, race, and belonging increasingly carried emotional weight, urgency, and moral implication. In this way, puberty functioned less as a topic of conversation than as a context that intensified the meaning of identity talk. One beginning puberty dyad illustrates how this intensification can unfold. The mother initiated the exchange with a familiar prompt asking what it means to be a girl. The daughter responded with uncertainty, “*I don't know*”, a response commonly observed earlier in puberty.

However, the mother treated this uncertainty not as a temporary placeholder but as a problem requiring correction, repeatedly reframing the question as something the daughter should already know, “*Yes you do. Don't ever say you don't know. You do know*”. In doing so, the interaction shifted from exploration toward expectation. As the conversation progressed, the stakes escalated. The mother moved across identity domains: gender, race, and gendered racial identity, layering normative messages about pride, strength, and understanding. These shifts implicitly framed puberty as a developmental threshold: the daughter was positioned as old enough to articulate and internalize identity meanings associated with bodily change. Identity talk thus became evaluative rather than exploratory.

The daughter's responses reflected this intensification. Her verbal engagement narrowed, affective distress increased, and embodied signals of overload emerged (“*I'm confused*,” “*I feel sick*”), accompanied by tears and withdrawal. These reactions suggested not resistance to identity itself, but difficulty managing the interpretive demands placed upon her in that moment. Puberty functioned here as an amplifier, heightening the emotional consequences of identity talk without necessarily expanding the space for coping or reflection. Importantly, this exchange does not suggest that puberty determines distress. Rather, it illustrates how pubertal transition can reorganize the meaning of identity questions, making them feel consequential, time‐sensitive, and morally charged. Identity talk that might have remained low‐stakes earlier became emotionally saturated once bodily change rendered identity more visible and socially legible. In this sense, puberty operates as a contextual intensifier, reshaping not only what is discussed but how identity is negotiated within the dyad.

## DISCUSSION

The present study examined how mothers and daughters co‐construct meanings of gender, ethnic‐racial identity, and their intersection during early adolescence, with particular attention to how pubertal status shapes these processes. The findings position identity development not as an internal cognitive achievement but as a relational process unfolding through interaction. Across dyads, puberty functioned as a contextual intensifier, heightening emotional salience, social comparison, and interpretive demand. Identity meanings were not simply expressed; they were initiated, scaffolded, negotiated, and stabilized through conversation. In this way, the study advances developmental theory by illustrating how embodied change becomes socially interpreted through family dialogue during a period of heightened vulnerability and possibility.

In the present study, puberty did not operate as a uniform developmental stage but as a contextual vulnerability that intensified girls' exposure to gendered and racialized meaning. As bodily changes became more visible, identity conversations increasingly incorporated themes of difference, comparison, and social evaluation. Girls moved from concrete descriptions of gender and race toward more emotionally nuanced and socially evaluative interpretations, particularly in advanced pubertal dyads. These patterns align with research showing that puberty heightens self‐consciousness and sensitivity to peer evaluation (Blakemore et al., 2010; Natsuaki et al., 2015), especially for girls navigating appearance norms and gendered expectations.

At the same time, the present study adds nuance by demonstrating how these interpretive demands emerge within real‐time family dialogue. Pubertal embodiment did not automatically produce identity complexity; rather, complexity arose when bodily change intersected with relational prompts, cultural narratives, and anticipatory discussions of social positioning. Importantly, vulnerability was not defined solely by biological change. Instead, it emerged through the intersection of bodily transformation with gendered and racialized contexts, including anticipated discrimination, appearance norms, and peer comparison. This finding echoes intersectional scholarship documenting how pubertal development can amplify racialized and gendered scrutiny, particularly for girls of color (Carter et al., 2017; Rogers et al., 2022). Rather than framing puberty as inherently risky, however, the present study suggests that puberty is interpretively demanding: a developmental moment in which meaning must be actively negotiated.

Maternal scaffolding emerged as a central coping resource within these conversations. Mothers initiated identity talk, expanded daughters' responses, reframed uncertainty, and calibrated emotional tone. These processes correspond closely to the interactional patterns identified in the results—expansion, refinement, and alignment—and extend research on ethnic‐racial socialization (Hughes et al., 2006; Umaña‐Taylor et al., 2014) and gender socialization (Hilliard & Liben, 2014). Some maternal messages appeared to resonate with broader cultural scripts of strength, independence, and resilience often discussed in relation to Black womanhood. Although the present study was not designed to analyze the Strong Black Woman schema (Abrams et al., 2014; Anyiwo et al., 2022) directly, these moments suggest that mothers may draw from culturally available narratives of strength as they help daughters interpret gendered racial positioning. Future research should examine when such messages function as affirming resources, when they may introduce pressure, and how girls themselves interpret these expectations during puberty.

Rather than focusing solely on the content of socialization messages, the present findings highlight how identity meanings are co‐constructed through dialogue. Mothers did not simply transmit cultural scripts; they engaged in responsive interaction that adjusted to daughters' developmental positioning. In earlier pubertal contexts, scaffolding tended to be more directive and normalizing, whereas in later contexts it became more dialogic and reflective. These patterns illustrate both reactive coping (constructing meaning in response to immediate conversational prompts) and protective coping (reframing potentially distressing topics). Importantly, daughters were not passive recipients of these processes. Their hesitation, humor, resistance, or elaboration shaped the trajectory of conversation, underscoring PVEST's emphasis on development as transactional. These findings extend ethnic‐racial and gender socialization literatures by showing that identity messages are not simply transmitted from parent to child. Instead, they are taken up, resisted, redirected, or emotionally contained within interaction. In this way, mother–daughter conversations operate as developmental exchanges in which both partners contribute to the meanings that become available, acceptable, or unresolved.

A central contribution of this study lies in identifying how conversational authority shapes identity stabilization. PVEST proposes that repeated coping responses consolidate into emergent identities. The present findings suggest that stabilization occurs not simply through repetition of messages but through the resolution of interactional authority within dialogue. In earlier pubertal contexts, maternal interpretations more often structured the conversation, reflecting daughters' developmental positioning and mothers' protective stance. As pubertal status advanced and identity talk became more evaluative, daughters increasingly asserted interpretive authority. Mothers shifted toward validation rather than definition, creating space for daughters' agentic meaning‐making. These shifts align with research documenting increasing autonomy and perspective‐taking during early adolescence (Steinberg & Morris, 2001) and clarify how such autonomy is negotiated in everyday interactions. Whose interpretation ultimately held within the conversation mattered because it shaped which meanings were reinforced, deferred, or left open. Identity stabilization, therefore, emerges not as an internal milestone but as a relational outcome.

Although race was not analyzed as a subgroup variable, intersectional dynamics were visible in the content and tone of conversations. As identity talk intensified across pubertal contexts, discussions increasingly incorporated structural awareness, historical references, and anticipatory social positioning. These patterns align with research documenting the emergence of critical consciousness among adolescents of color (Leath et al., 2023). By integrating PVEST with intersectionality, the present study highlights how vulnerability and coping are shaped by social location. Puberty does not unfold in a social vacuum; it intersects with systems of gendered and racialized power. Some dyads explicitly named these dynamics, while others remained at more descriptive levels. This variability underscores PVEST's emphasis on phenomenology: identity development depends not only on social conditions but on how youth interpret and are supported in interpreting their lived experiences.

### Theoretical contributions

This study makes three primary contributions to developmental science. First, it reconceptualizes puberty as an interpretive intensifier rather than a deterministic risk factor. Pubertal status heightened the salience of identity conversations but did not determine their direction. This framing aligns with calls to move beyond deficit‐oriented models of early development (Carter & Seaton, 2025). Second, the study extends PVEST by demonstrating how coping processes are enacted interactionally within the family microsystem. Rather than treating coping as an individual trait, the findings show how coping resources are co‐produced through dialogue. Third, the dyadic analytic approach highlights the methodological value of examining conversational processes. Identity development is often studied through surveys or individual interviews; by contrast, the present study reveals how identity meanings are constructed, negotiated, and stabilized within relational exchange.

### Limitations and future directions

Several limitations warrant consideration. The structured nature of the dialogue task may have shaped conversational dynamics. Although pubertal status was central to the analysis, longitudinal data would help clarify how repeated conversational patterns contribute to identity stabilization over time. In addition, the study did not conduct subgroup comparisons across sociocultural contexts. The present analysis also prioritized interactional processes over a fuller thematic analysis of identity content. As a result, we did not systematically examine specific cultural narratives, such as strength, resilience, respectability, beauty, or protection, that may have shaped dyadic meaning‐making. Future work could extend these findings by examining the content of gendered ethnic‐racial identity messages, including how girls interpret culturally salient messages about Black girlhood and Black womanhood. Lastly, future research could also build on this qualitative dyadic approach by coding conversational features quantitatively, including turn‐taking, response latency, maternal elaboration, youth resistance, repair, and closure. Such analyses could help clarify how specific interactional patterns shape identity meaning‐making across time. Future research could examine how dyadic identity processes vary across families, as well as in conversations involving fathers, caregivers, or peers.

## CONCLUSION

Identity development during puberty emerges not as a solitary cognitive process but as a relational achievement shaped through everyday dialogue. By centering dyadic interaction and grounding interpretation in PVEST, the present study illustrates how embodied change becomes socially mediated meaning. Puberty intensifies interpretive demands, but identity is stabilized through negotiation, scaffolding, and shifting authority within family relationships. Early adolescence, therefore, represents not only a period of biological transformation but also a time of collaborative identity construction. Taken together, these findings suggest that puberty should be conceptualized not only as a biological transition but also as a relational interpretive turning point. As bodily change becomes socially visible, identity questions acquire heightened meaning, prompting families to engage in conversations that interpret what those changes signify within gendered and racialized contexts. Through these exchanges, identity meanings are scaffolded, contested, and gradually stabilized. In this way, early adolescence represents a period in which identity is not simply discovered but collaboratively authored within the family microsystem.

These findings suggest that supportive identity development during puberty depends less on delivering prescriptive messages and more on engaging in responsive, developmentally attuned dialogue. Conversations that allow space for uncertainty, humor, hesitation, and revision may foster more adaptive identity integration than rigid or premature evaluation. For practitioners and educators, this highlights the importance of creating relational contexts where identity can be explored without immediate closure. For families, it suggests that authority can gradually shift toward youth without relinquishing guidance, supporting both protection and autonomy during early adolescence.

## AUTHOR CONTRIBUTIONS

**Joonyoung Park:** Writing – review and editing; conceptualization. **Ha Bui:** Writing – review and editing; conceptualization. **Rona Carter:** Conceptualization; funding acquisition; writing – original draft; methodology; writing – review and editing; investigation; formal analysis; project administration; data curation; supervision. **Jessica Pitts:** Conceptualization; writing – review and editing. **Julia Cross:** Writing – review and editing.

## FUNDING INFORMATION

Institute for Research on Women and Gender, University of Michigan G024491.

## CONFLICT OF INTEREST STATEMENT

We have no known conflict of interest to disclose.

## ETHICS STATEMENT

This study was approved by the Institutional Review Board at the University of Michigan (IRB #HUM00183043; approval date: January 16, 2022). All study procedures complied with institutional guidelines for research involving human participants.

## CONSENT

Written informed consent was obtained from parents or legal guardians of all participating youth. Youth participants provided written assent prior to participation in the study.

## Data Availability

The data that support the findings of this study are available from the corresponding author upon reasonable request.
